# Community Structure and Activity of a Highly Dynamic and Nutrient-Limited Hypersaline Microbial Mat in Um Alhool Sabkha, Qatar

**DOI:** 10.1371/journal.pone.0092405

**Published:** 2014-03-21

**Authors:** Roda Al-Thani, Mohammad A. A. Al-Najjar, Abdul Munem Al-Raei, Tim Ferdelman, Nguyen M. Thang, Ismail Al Shaikh, Mehsin Al-Ansi, Dirk de Beer

**Affiliations:** 1 Department of Biological and Environmental Sciences, Qatar University, Doha, Qatar; 2 Max-Planck Institute for Marine Microbiology, Bremen, Germany; 3 Environmental Studies Center, Qatar University, Doha, Qatar; University of Auckland, New Zealand

## Abstract

The Um Alhool area in Qatar is a dynamic evaporative ecosystem that receives seawater from below as it is surrounded by sand dunes. We investigated the chemical composition, the microbial activity and biodiversity of the four main layers (L1–L4) in the photosynthetic mats. Chlorophyll *a* (Chl *a*) concentration and distribution (measured by HPLC and hyperspectral imaging, respectively), the phycocyanin distribution (scanned with hyperspectral imaging), oxygenic photosynthesis (determined by microsensor), and the abundance of photosynthetic microorganisms (from 16S and 18S rRNA sequencing) decreased with depth in the euphotic layer (L1). Incident irradiance exponentially attenuated in the same zone reaching 1% at 1.7-mm depth. Proteobacteria dominated all layers of the mat (24%–42% of the identified bacteria). Anoxygenic photosynthetic bacteria (dominated by Chloroflexus) were most abundant in the third red layer of the mat (L3), evidenced by the spectral signature of Bacteriochlorophyll as well as by sequencing. The deep, black layer (L4) was dominated by sulfate reducing bacteria belonging to the Deltaproteobacteria, which were responsible for high sulfate reduction rates (measured using ^35^S tracer). Members of Halobacteria were the dominant Archaea in all layers of the mat (92%–97%), whereas Nematodes were the main Eukaryotes (up to 87%). Primary productivity rates of Um Alhool mat were similar to those of other hypersaline microbial mats. However, sulfate reduction rates were relatively low, indicating that oxygenic respiration contributes more to organic material degradation than sulfate reduction, because of bioturbation. Although Um Alhool hypersaline mat is a nutrient-limited ecosystem, it is interestingly dynamic and phylogenetically highly diverse. All its components work in a highly efficient and synchronized way to compensate for the lack of nutrient supply provided during regular inundation periods.

## Introduction

In nature, microorganisms tend to aggregate forming assemblages of different levels of complexity ranging from mono-species biofilms to well-defined laminated microbial mat ecosystems [1], [2]. These arrangements provide better defensive capabilities as well as efficient transfer of resources and energy from one layer to the other. Microbial mats are complete ecosystems on sub-millimeter scale that colonize extreme habitats [2], [3]. Indeed, developing adaptations that enabled the microorganisms in the mats to proliferate in such habitats is a surplus as the extreme conditions exclude the presence of higher organisms that may compete with them. Microbial mats provide several important ecological roles especially to the marine intertidal ecosystem [4]. In addition to their contribution to the global primary productivity and CO_2_ fixation, these communities interact with the sediment, “glue” particles, and enhance precipitation and lithification [2], [5], [6]. Within a microbial mat, species distribution and abundance are strongly influenced by the physical and chemical properties. The physical conditions include light, temperature, and pressure, whereas the chemical parameters are oxygen, pH, redox potential, salinity, and available electron acceptors and donors [2].

Microbial mats are highly active ecosystems that accommodate a great diversity of species, variable resources and a wide range of metabolic processes. The activities of photosynthetic microorganisms (oxygenic and anoxygenic), aerobic heterotrophs, and sulfate reducers in addition to mass transfer limitation lead to the formation of distinct microenvironments. These dynamic microenvironments vary on both spatial and temporal scales providing heterogeneous environments leading to diverse community structures.

Because of limited ability to isolate the majority of microorganisms, the application of culture-independent approaches to the study of microbial ecology, in general, and in microbial mats in particular has expanded our understanding to this field [7]. There are many studies in the literature regarding phylogenetic studies based on 16S rRNA gene sequences, which have identified the major phylotypes and the dominant species [8]–[10]. They also resolve vertical variation in microbial community structure within the mat. Typically, the upper layer is dominated by oxygenic photosynthetic microorganisms (i.e., cyanobacteria and diatoms), whereas the deeper layers are characterized by anoxygenic sulfur bacteria, heterotrophs and sulfate reducing bacteria. Therefore, this has reinforced the idea that microbial mats have great species diversity with a wide range of metabolic capabilities, which makes them ideal models to study complete ecosystem. Other studies emphasized the spatial variations of the function with the depth. Oxygenic phototrophs are in the upper layer because of the suitable light environment (quantity and quality), whilst anoxygenic photosynthesis takes place in deeper layers, where both electron donor (i.e., sulfide) and suitable wavelengths (far-red spectra) are available. Apparently, underneath these layers, there is no light and thus heterotrophic mode of life is dominant. With the recent development of suitable methods, it is now possible to track the spatial variations in the activity within the oxic layer at the sub-millimeter scale [11]. Using light and oxygen microsensors in the same spot, it is possible to measure the absorbed light energy and the chemical energy stored in photosynthesis in each layer. Thus, variations in the efficiency of photosynthesis between layers in the euphotic zone can be calculated [12], [13]. However, combined approaches (microbial community structure and activity) are considered optimal approaches to better understand how microbial mat ecosystem function and individual community metabolism.

**Figure 1:**
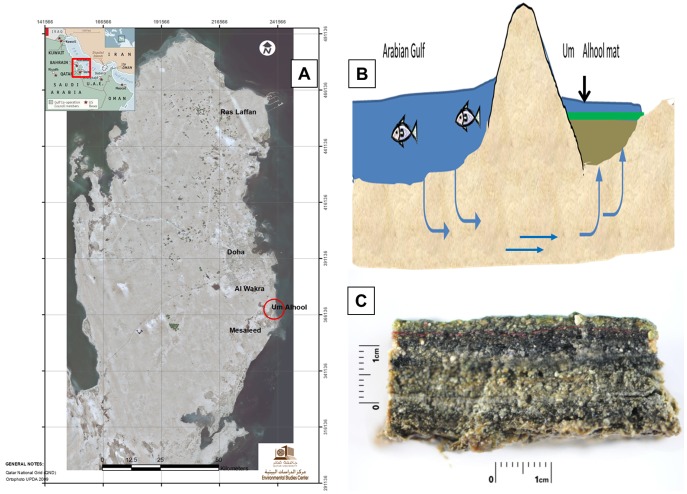
Location of Um Alhool mat, which is part of Mesaieed Sabkha in the South-East of Qatar (A). The separation of Um Alhool ecosystem from the seawater by sand dunes is shown depicted in the schematic sketch in (B). The cross section of the stratified layers in the Um Alhool mat are shown in (C).

The coastal ecosystems of Qatar and those of the neighboring Gulf countries are found in one of the most arid regions of the world. These coastal ecosystems are fragile and threatened by human activities such as urban development, pollution, oil and gas mining and uncontrolled recreation. The coastline in Qatar is generally flat, and mangroves are locally a prominent feature. Coastal Sabkha are predominantly found on the eastern coast of Qatar, with the largest site located in the southeast, immediately south of Mesaieed Sabkha. Mesaieed is the largest coastal Sabkha in the country, extending over 55 km in length and between 4 and 14 km in width. Apart from saline crust, Mesaieed Sabkha is characterized by high gypsum content in the subsurface layers. The texture of these layers can be characterized as sandy clay-loam. Um Alhool is part of Mesaieed Sabkha and is located between Al Wakra and Mesaieed at south east of Qatar (see the map in Fig. 1). In addition to mangroves, the biota of Mesaieed Sabkha comprises microbial mats.

Um Alhool mat ecosystem is very interesting, as it is permanently inundated with sea water in winter, although there is no direct contact with the sea water. However, the ecosystem is supplied with sea water from the bottom (Fig. 1B). These conditions may raise questions concerning the activity and biodiversity of the microorganisms inhabiting this ecosystem. Therefore, in this study we aimed to answer the following questions: (i) To what extent is the primary productivity of Um Alhool mat comparable to other hypersaline mats?, (ii) What is the bacterial and eukaryotic biodiversity in the mat under such conditions of limited surface water flow?, and (iii) What is the contribution of oxygenic respiration versus sulfate reduction in organic material degradation? We wanted to test the hypothesis that Um Alhool mat might compensate for nutrient limitation, which is normally supplied through surface water flow in other microbial mat systems, by highly active organic material degradation carried out by possibly high biodiversity. Thus, highly active nutrient remineralization will provide fast and continuous supply to the producers in the upper layers. In turn, photosynthetic microorganisms provide the ecosystem with organic materials required by them. Therefore, we investigated the bacterial and eukaryotic community structure of Um Alhool mat using 454 pyrosequencing of 16s and 18s rRNA genes and measured their activity (i.e., photosynthesis and sulfate reduction as major organic material processes in microbial mats). We quantified the pigment composition for each layer through the mat using HPLC and hyperspectral imaging. We measured the photosynthetic activity and microscale light distribution using microsensors as well as the chemical composition and sulfate reduction rates at different layers.

## Materials and Methods

### Study site and sampling

Um Alhool is part of Mesaieed Sabkha, which is the largest Sabkha in Qatar. It is localized at south east of Qatar (Fig. 1A). The Um Alhool mat system consists of 2–3 separated mats; each of which extends over an area of approximately 60 m^2^ and all of them are separated permanently from seawater by big sand dunes. In winter the mats are covered continuously with sea water (salinity 5%) that seeps from the adjacent Arabian Gulf water from below in addition to limited input from rain (never exceeds 100 mm y^−1^). The mat was covered by approximately 30 cm of sea water with water temperatures ranging between 15–26°C in December; however, water temperature can reach more than 33°C (air temperature > 47°C) in summer. The mat is also inundated with seawater in summer, but it is shallower and has higher salinity (>13%) than in winter because of high evaporation rates.

Microbial mat samples (10×20 cm) were collected in December 2011. Several mat pieces were taken from the field, transported in the ambient sea water to the Environmental Study Center (ESC), Qatar University, and kept under simulated *in situ* conditions. All the required permissions for visiting the site and taking samples were issued from ESC, who is partner in this work. No sampling for vertebrates was done in this study. Microsensor measurements and DNA extraction were performed on those samples one day after collecting them. Additional mat samples were sent to the Max-Planck Institute for Marine Microbiology, Germany, where they were incubated under similar conditions (temperature of 26–28°C, salinity of 5%). The mat pieces were kept under a 10-h light–14-h dark illumination regime at incident irradiance of approximately 480 μmol photon m^−2^ s^−1^ with a spectral composition similar to sunlight. Pigment analysis and sulfate reduction rates were conducted in the Max-Planck Institute for Marine Microbiology.

### Chemical analysis

Surface and pore waters were collected from the study sites in December 2011. Pore waters were taken at depths of 1, 3, 5, 8, 10, 13, and 15 cm below surface with about 20-cm-long pore water zippers made of stainless steel lances connected to plastic syringes via 3-way valves and immediately filtered through 0.45-μm disposable membrane filters into pre-conditioned different sampling vials: (i) samples were collected in plastic centrifuge tubes containing 5% zinc acetate solution for sulfate and sulfide, (ii) acid pre-cleaned glass exetainers for dissolved inorganic carbon, and (iii) tubes without additions were used for nutrient samples (see [14]). Aliquots were either kept frozen or kept in the dark at 4 °C until further analysis. Total sulfide was measured in the zinc acetate-preserved pore waters using photometric measurements according to Cline [15]. After centrifugation, the determination of dissolved sulfate was carried out from the same aliquots using a Dionex LC30 DX 500 ion chromatograph.

Silicate, phosphate, ammonium, nitrate, and nitrite were spectrophotometrically quantified with a Skalar continuous-flow-analyzer according to methods previously described [16]. Salinity of filtered samples was measured with a refractometer (precision: ±0.3). Total and dissolved inorganic carbon were analyzed with TOC/TNb Analyzer, a multi N/C® 2100S Thermo catalytic oxidation, and MC-NDIR detection for TOC analysis. Water content of sediment samples was measured by weighing the sediment before and after drying and sediment porosity calculated. The water temperature was measured with a digital sensor [ama-digit ad 20th (−50 to +300°C) digital thermometer] that has in situ precision of 0.5°C.

### Pigment analysis

Pigments concentrations were semi-quantitatively measured using hyperspectral reflectance imaging of the intact mat piece and quantitatively using High Pressure Liquid Chromatography (HPLC) on pigment extracts. Hyperspectral imaging and photopigment identification from the hyperspectral images were performed as previously described [17], [18]. Chl *a* in the mat was approximated from the hyperspectral image by calculating the hyperspectral microphytobenthos index (MPBI) [18] as follows:
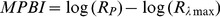
(1)


where *R_λmax_* is the measured reflectance at *λ_max_* and *R_p_* is the value of the fitted linear trend in the NIR range (720–800 nm) extrapolated to λ_max_.

The uppermost 6 mm part of the mat were sliced in 0.5-mm thick layer for the first 3 mm and 1 mm for the rest using cryomicrotome (Mikrotom-Kryostat HM 505E, Mikrom, USA) at −36 °C. Pigments were extracted by adding 1 ml cold acetone (100%) on top of the freeze-dried slices, which were then incubated in sonication water bath for 3 min, and then they were kept in dark at −20 °C overnight. Subsequently, the supernatant was filtrated through a 0.45-μm Acrodiscs CR 4-mm syringe filter (Pall Gelman Laboratory, USA) and the filtrates were injected into a reverse-phase HPLC consisting of a Waters 996 photo diode array detector (PDA) and a Waters 2695 separation module (Waters, MA). Pigments were separated on the basis of the method described by Wright et al. [19]. Identification and quantification were done by comparing the retention time and absorption spectrum of the eluents with those of pigment standards. For bacteriochlorophyll identification, because a standard of mixed bacteriochlorophylls extracted from *Rhodopseudomonas sphaeroides* (Sigma–Aldrich, Germany) was used, this pigment was quantified only in arbitrary units. The scytonemin standard was from Sigma-Aldrich, Germany and the other standards are from DHI Water and Environment, Denmark.

### Microsensor measurements

A microbial mat sample was placed in a small flow-chamber (11×4.5×5 cm) connected with plastic tubing fixed to a submersed water pump to maintain steady flow of seawater (salinity 5%) above the mat surface. The flow cell was fixed on a holder and placed under a vertically incident collimated light beam from a tungsten-halogen lamp (KL 2500, Schott).

High spatial resolution measurements of dissolved O_2_ concentration were performed with a fast response Clark-type microelectrode (tip diameter approximately 10−20 μm; [20]). The oxygen microsensor was linearly calibrated using 2-points calibration (in the anoxic layer of the mat and in the aerated overlaying seawater) after applying temperature- and salinity-corrected O_2_ solubility [21]. Rates of net photosynthesis (P_N_ in μmol O_2_ m*^−^* ^2^ s^−1^, calculated from steady-state profiles of O_2_), as well as volumetric rates of gross photosynthesis (P_G_ in μmol O_2_ m*^−^* ^3^ s^−1^, measured by the light–dark shift method [22]), were conducted at two incident irradiances of 130 and 530 μmol photon m*^−^* ^2^ s*^−^* ^1^. P_G_ measurements were conducted in vertical depth intervals of 100 μm, with 3 replicates at each depth. The illumination at each irradiance level was kept constant for up to 30 minutes to reach steady state O_2_ conditions, which was determined from the microsensor signal before each measurement.

Light attenuation in the mat was measured using a fiber-optic scalar irradiance microprobe (80 μm) [23] connected to a spectrometer (USB4000, Ocean Optics, Dunedin, FL, USA). Scalar irradiance micro-profiles were measured in three different spots, normalized at each wavelength to the scalar irradiance at the mat surface, and averaged. The spectral irradiance reflectance of the mat (R_λ_) was calculated as R_λ_  =  I_λ,mat_/I_λ,ref_, where I_λ,mat_ and I_λ,ref_ are the back-scattered spectral radiances measured above the mat and a white reflectance standard (Spectralon; Labsphere, North Sutton, NH, USA), respectively, with a fiberoptic field radiance microprobe [24]. The downwelling incident irradiance (I in μmol photon m^−2^ s^−1^) was measured by a PAR quantum irradiance sensor (LI-190 Quantum) connected to a light meter (LI-250, both from LI-COR Biosciences, Lincoln, NE, USA).

To prevent self-shading and allow simultaneous measurements, the O_2_ and scalar irradiance microsensors were positioned at zenith angles of 135° and *−*135°, respectively, relative to the vertically incident light. The measuring tips of both microsensors were positioned at the mat surface and in close proximity to each other using a 3-axis manual micromanipulator aided by observation under a dissection microscope (SV6, Zeiss).

### Sulfate reduction measurements

Microbial sulfate reduction rates were measured using the whole-core incubation technique by the incubation of short sediment cores (2.6 cm diameter) after the injection of 20 μl of carrier-free ^35^S-labeled sulfate (≈50 kBq) in each layer (1 cm thick) as previously described [25]. Briefly, incubations were done in the dark for 6.5 h. The incubation was stopped by transferring the mat slices into 50-mL plastic centrifuge tubes containing 20 mL zinc acetate (20%, v/v). The total amount of ^35^S-labeled-reduced inorganic sulfur was determined using the single-step cold distillation method of Kallmeyer et al. [26] by counting on a Tricarb 2500 liquid scintillation counter. Sulfate reduction rates (nmol cm^−3^ d^−1^) were calculated using the equation described in [27].

### DNA extraction and 454 Pyrosequencing

Distinct layer of the microbial mat, based on color (2–3 mm), were sliced using sterile scalpels after bringing the samples from the field to the lab. DNA was extracted and purified from each layer using the UltraClean soil DNA isolation kit (MO-BIO Laboratories, Inc., Carlsbad, CA, USA) according to the manufacturer’s instructions.

The extracted DNA from the layers was then analyzed by 454 pyrosequencing for bacterial, archaeal and eukaryotic diversity. Primer sets (27Fmod AGRGTTTGATCMTGGCTCAG and 519Rmodbio GTNTTACNGCGGCKGCTG) were used for bacterial sequences, (archea349F GYGCASCAGKCGMGAAW and archaea806R GGACTACVSGGGTATCTAAT) for archaeal sequences, and (Euk7F AACCTGGTTGATCCTGCCAGT and Euk570R GCTATTGGAGCTGGAATTAC) for eukaryotic sequences. Pyrosequencing was performed by Research and Testing Laboratories, Lubbock, Texas, using a Roche 454 FLX Genome Sequencer system as previously described by Dowd et al. [28].

### Sequence analysis

Diversity and community structure analyses were performed on 18078 bacterial, 9299 archaeal, and 22651 eukaryotic sequences obtained from different layers of the studied mat. Sequence analysis was performed as described in [29] and [30]. The quantitative information (number of individual reads representing a taxonomic path) was obtained by mapping taxonomic path of each cluster reference to all reads within the corresponding cluster as well as to their corresponding replicates. This step allowed, minimizing the bounds of PCR and pyrosequencing biases. To confirm the taxonomic affiliation of the sequences, all cluster references were imported into ARB [31] and inserted into the guide tree of the SILVA SSURef dataset (release 108) (More details can be found in the “Materials and Methods” in File S1). The Archaeal primer additionally amplified bacterial false-positives, which were excluded from the final calculations regarding Archaea.

## Results

### Mat structure

**Figure 2:**
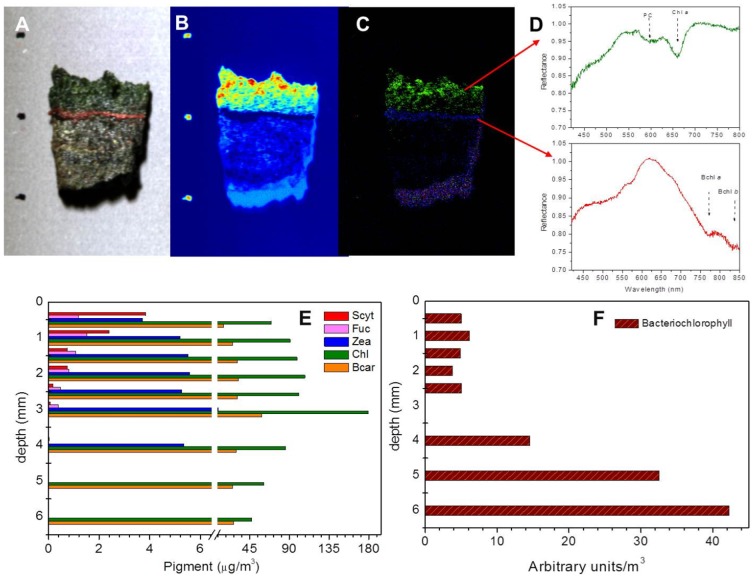
Vertical distribution of photopigments in a representative piece of the Um Alhool mat shown in true color image (A), using hyperspectral imaging and HPLC. Panel (B) represents the false color image of Chl *a* distribution calculated using MPBI. The color code ranges from blue (less Chl *a*) to red (high Chl *a*). Composite RGB image (C) shows distributions of Chl *a* (green channel), phycocyanin (PC; red channel) and bacteriochlorophyll *a* (BChl *a*; blue channel), as derived from hyperspectral imaging based on their characteristic spectral signatures (D). Scale bar is 1 cm. The lower panel shows the vertical distribution and the concentration (in μg m^−3^) of photopigments (Scytonemin, fucoxanthin, zeaxanthin, chl, and β-carotene) measured using HPLC (E), and the distribution of Bacteriochlorophyll (F) calculated as arbitrary units.

The mat exhibited conspicuous vertical stratification, with a characteristic green layer (L1) of variable thickness (1–2 mm) at the mat surface (see Fig. 1C & true color images in Fig. 2). This distinct appearance was mainly due to cyanobacteria as indicated by the presence of pronounced absorption at the wavelengths of maximal absorption by chlorophyll *a* (675 nm) and phycocyanin (625 nm) detected by hyperspectral imaging (Fig. 2D). However, the presence of algae and diatoms in that layer cannot be excluded, especially because of the presence of intense Chl a signal (Fig. 2B), which is a general pigment found in all oxygenic photosynthetic organisms. Chl a was unevenly distributed in the euphotic zone, with “hot spot” patches close to the surface and down to 1 mm below the surface. Furthermore, weak Chl a signals (light blue color in hyperspectral image; Fig 2B) were scattered in deeper layers, indicating the presence of buried Chl a.

Directly underneath the green layer, a brown layer (L2) of ∼1.5 mm followed by a very thin (∼1 mm) red layer (L3). The latter layer was permanently present in the whole mat ecosystem and not just in this specific piece. The red layer is characterized by the presence of anoxygenic phototrophic microorganisms evidenced by pronounced absorption at the wavelengths of 778 and 830 nm (i.e., absorption maxima for bacteriochlorophylls; Fig. 2D). The fourth layer (L4) was thicker than the upper layers (6–8 mm) and had a gray to black color, presumably due to metal-sulfidic precipitates. A relatively thick layer of light colors (yellow to green) was found again below L4, followed by thin gray layer and finally black layer again, suggesting annual burial event.

In addition, the mat ecosystem has ample eukaryotic microorganisms and mesofauna that can be seen by microscope and by eyes, respectively. For example, several worms and small marine arthropods were seen at the surface of the microbial mat in the morning, because of O_2_ limitation at deeper layers during night (more details below).

### Chemical composition

**Figure 3:**
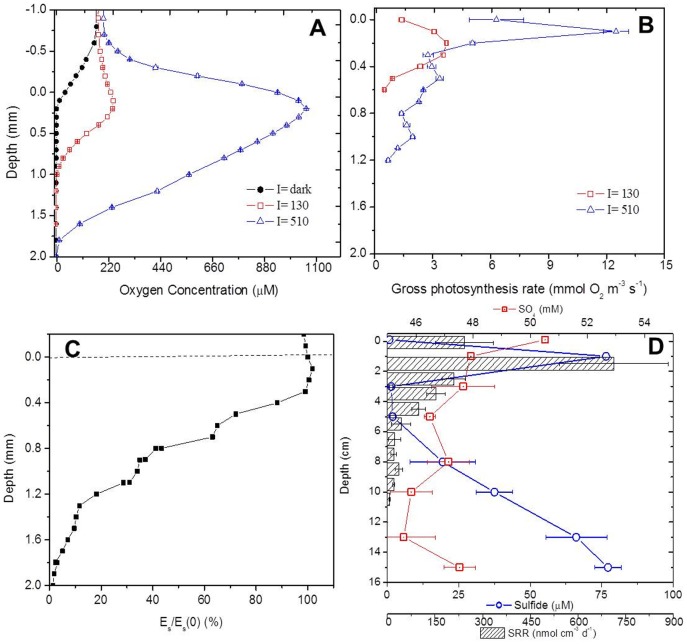
Vertical distribution of activity (i.e., photosynthesis and SR) and incident irradiance inside the studies mat. The upper panel shows steady state O_2_ profiles inside the mat (A) in dark (filled circles), and at incident irradiances of 130 and 510 μmol photon m^−2^ s^−1^ (empty squares and triangles, respectively), while (B) represents vertical distribution of rates of gross photosynthesis (in mmol O_2_ m^−3^ s^−1^) measured in light/dark shift method. Vertical profiles of scalar irradiance integrated over PAR and were normalized to the value measured at the mat surface, and are plotted in linear scale (C). Sulfate reduction rates (in nmol cm^−3^ d^−1^) and the vertical distribution of sulfate and sulfide in the mat (D), note that depth is in cm in (D).

Potassium and phosphate concentrations in Um Alhool mat were higher than those characterizing marine water. Potassium and phosphate concentrations in Um Alhool mat were 20 and 0.8 μM, respectively, compared with those of 10 and 0.53 μM in seawater (Table 1). However, other ions (i.e., magnesium, sodium, and lithium), nutrients (i.e., ammonium, silicate, and nitrate), and dissolved organic and inorganic carbon in the studied mat showed lower concentrations than those in seawater and lake water. Sulfate concentration in surface water of the studied mat was also less (Fig. 3D) than that documented in other microbial mats. However, within the mat, sulfate concentration was very similar in the pore water collected from different layers in the mat (Fig. 3D).

**Table 1:** Comparison between ion and nutrients contents of the seawater overlaying Um Alhool mat and seawater of the open ocean and the one covering the mat of Cheprana in Spain.

	Um Alhool	Seawater^a^	Chiprana lake water
**in mM**			
**K**	20	10	5^c^
**Mg**	85	55	322^c^
**Na**	690	486	497^c^
**inμM**			
**Li**	53	26	-
**NH_4_**	13.89	-	85.5^d^
**NO_3_**	0.98	6.94	20.9^d^
**PO_4_**	0.82	0.53	0.4^d^
**Silicate**	13.77	0.54	85.4^d^
**in mg/L**			
**DIC**	12.74	32.8– 81.9	-
**DOC**	23.69	70^b^	-

a [32].

b [49],

c [34], ^d^ [50].

### Functional characterization

#### 1. Pigment analysis

Pigment analysis, which was performed on the upper 6 mm sectioned in 0.5–1 mm thick layers (Fig. 2E&F), supported the hyperspectral measurements with respect to Chl *a* and bacteriochlorophylls. Chl a was detected in all mat layers (Fig 2E), with highest concentration in the layer at 3 mm and the lowest concentration in the layer at 6 mm (∼ 180 and 45 μg m^−3^, respectively). Bacteriochlorophyll concentration (Fig 2F) was lowest in the upper 2 mm of the mat, but progressively increased with the depth until reaching the highest concentration in the deepest layer (at 6 mm).

As expected, the concentration of other accessory and protective pigments (Fig 2E) decreased, with the depth except for β-carotene, which showed similar concentrations. The concentration of Scytonemin (UV-scavenging pigment), was highest at the surface (3.83 μg m^−3^) and gradually decreased with the depth, until it was below the detectable limit of <4 mm. The highest concentration of fucoxanthin (proxy pigment for diatoms and brown algae), was found 1 mm below the surface, and then steeply decreased until it was below the detection limit at 4 mm. Zeaxanthin, an indicator for cyanobacteria, was detected in the upper 4 mm of the mat (5.34 μg m^−3^), and it was not found in the deeper parts of the studied mat. Its concentration was similar in the layers, where it was detected (5.10 ± 0.70 μg m^−3^), except for the layer at 3 mm that showed the highest concentration (9.4 μg m^−3^), which also showed the highest Chl a concentration. This indicates that cyanobacteria were found in the upper 4 mm of the mat, and they were mainly concentrated in the layer at 3 mm from the surface. On the other hand, β-carotene concentration remained similar between the layers (27.1±5.4 μg m^−3^) except in the layer at 3 mm, where it was two times higher (∼ 59 μg m^−3^) than that in other layers.

#### 2. Light attenuation and oxygenic photosynthesis

A small fraction of incident irradiance was back-reflected from the mat surface (1.2%), whereas the rest (98.8%) was absorbed in the euphotic zone of the mat (i.e., euphotic zone). Light exponentially attenuated below 0.3 mm in the mat at attenuation coefficient 3.76 mm^−1^. Directly below the mat surface, incident irradiance was slightly enhanced (105%) because of high light scattering; conversely, only 1% of the incident irradiance (relative to that at the surface) was measured at 1.7 mm. Interestingly, light profiles showed regions of sudden local light attenuation (at 0.6 and 1 mm) that may correspond to the spots of high photosynthetic activity.

Oxygen concentration rapidly increased as incident irradiance increased (Fig 3A&B). The peak of O_2_ concentration maxima at incident irradiance 130 μmol photons m^−2^ s^−1^ emerged directly below the surface (100 μm). Oxygen concentration increased to slightly more than saturation in the overlaying water (240 μM versus 200 μM), indicating limited photosynthetic activity. This was evidenced by measuring rates of volumetric gross photosynthesis using light–dark shift method (Fig.3B), which showed a small peak of activity (∼ 3 mmoL O_2_ m^−3^ s^−1^) at 200 μm, whereas the active euphotic zone extended over 500 μm.

When incident irradiance increased ∼4 times (510 μmol photon m^−2^ s^−1^), O_2_ profile expanded over deeper region in the euphotic zone, in addition, O_2_ concentration reached a maximum of 1056 μM (i.e., ∼4 times higher than it was at an incident irradiance of 130 μmol photons m^−2^ s^−1^). Furthermore, this not only increased the rates of gross photosynthesis but also the depth of euphotic zone (1.2 mm) and number of peaks of activity (n  =  3). The rate in the first peak, which was directly below the surface, pronouncedly increased to 12.5 mmol O_2_ m^−3^ s^−1^ (Fig. 3B). A second and a third peak emerged at 0.5 and 1 mm, respectively. In dark, the mat ecosystem became anoxic even in the top layers, and rate of respiration was −0.255±0.065 μmol O_2 _m^−2^ s^−1^. The respiration rates for the mat ecosystem during light periods, measured as the difference between rates of gross and net photosynthesis, increased with increasing incident irradiances (see Fig.S1 in File S1).

#### 3. Sulfate reduction rates

The integrated areal rates of sulfate reduction over a depth of 10 cm of the mat and those of the sediment underneath reached to 1558.4±426.2 nmol cm^−2^ d^−1^. Sulfate reduction activity peaked in the 1–2 cm layer (713 nmol cm^−2^ d^−1^; Fig. 3D). This coincided with steep sulfate consumption and a peak of sulfide production at the same layer. The uppermost layer (i.e., upper 1 cm, where the active microbial mat is located) exhibited approximately 3-fold lower sulfate reduction (SR) activity with a rate of 241.6 ± 91.2 nmol cm^−3^ d^−1^ (2.77 μmol m^−3^ s^−1^). Below the peak of SR activity, SR rates decreased progressively until 7 cm, where it slightly increased again (35.8 versus ∼ 20 nmol cm^−3^ d^−1^ in the upper layer). In the same layer, pronounced increase of sulfide emergence appeared after being close to zero in the upper 2 cm. In the deeper layers, both sulfate and sulfide progressively increased, without any considerable effect from SR, suggesting a deeper feeding source(s) of sulfate and sulfide.

### Diversity of different communities in the mat

In total, we investigated 8360 bacterial-, 2915 archaeal-, and 1484 eukaryotic-aligned sequences (Table 2). From which a considerable proportion, especially in archaeal domain, showed less than 80% identity to the best hits, suggesting potential novel lineages in the studied microbial mat.

**Table 2:** Total number of aligned sequences of bacteria, archaea, and eukaryotes in each layer of the studied mats.

	Bacteria	Archaea	Eukaryotes
S50L1	1779 (8.5)	911 (27.4)	442 (11.3)
S50L2	2045 (10.8)	881 (29.4)	239 (9.6)
S50L3	2926 (19.5)	775 (29.4)	219 (12.8)
S50L4	1610 (29.5)	348 (30.0)	584 (10.6)
**Sum**	**8360**	**2915**	**1484**

The number between the parentheses represent the percentage of potential new lineages (to the total number of sequences) on the phylum level (<80% identity to the best hit in NCBI).

#### 1. Bacterial community analysis

**Figure 4:**
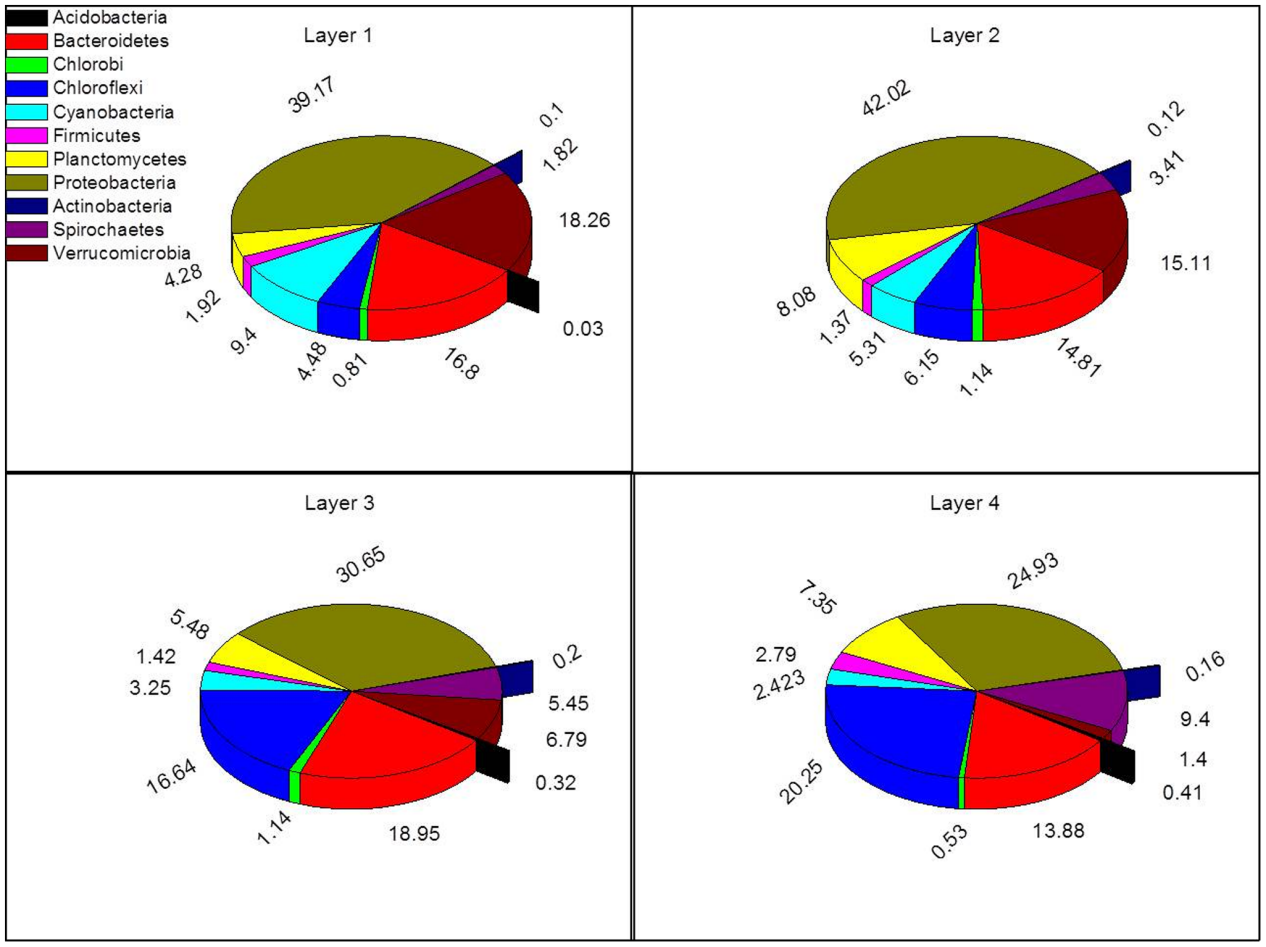
Pie charts representation of the abundance of different bacterial phyla distributed in layers of the studied mat.

Diversity and abundance of different members of the main phyla in the bacterial domain showed clear variations with depth (Fig. 4). Proteobacteria had the highest sequence frequency between all the bacteria investigated in each layer (∼ 25%–43%) of the studied mat. Their sequence frequency in the upper 2 layers (L1 and L2) was the highest (40%–42%), but then it decreased to reach 25% in L4. The sequence frequency of cyanobacteria was the highest at the uppermost layer (9.4% in L1) and gradually decreased with depth to reach only 2.4% in L4. This distribution is expected as active oxygenic phototrophic microorganisms tend to stay closer to layers, where light penetrates. Similar trend was also found for members of the phylum Verrucomicrobia, as they dominated L1 (18.3%) and this dominancy rapidly decreased to 1.4% in L4. Conversely, the sequence frequency of members belonging to the phyla Chloroflexi and Spirochaetes both progressively increased with depth. In L1, they were the lowest (∼ 4.5% and 1.8% for Chloroflexi and Spirochaetes, respectively), whereas their sequence frequency increased in L4 to reach 20% and 9.4%, respectively. Members of the phyla Planctomycetes and Firmicutes showed no pattern in sequence frequency present between the different mat layers. Microorganisms belonging to phylum Chlorobi had the sequence frequency of approximately 1% in L2 and L3, 0.8% in L1, and lowest in L4 (0.5%). Members of the phylum Acidobacteria were abundantly found in the deeper two layers (L3 and L4) and were not detected in L1 and L2.

**Figure 5:**
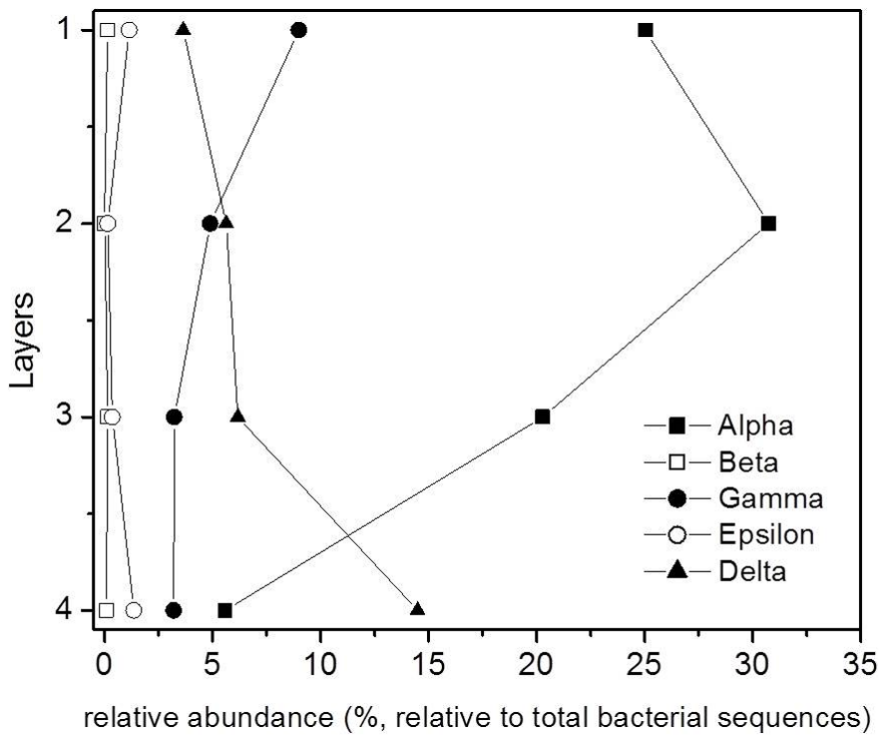
Vertical distribution through mat layers of relative abundance (relative to the total identified bacterial sequences) of different Proteobacteria classes; Alpha (filled square), Beta (opened square), Gamma (filled circle), Epsilon (opened circle) and Delta (filled triangle).

In-depth analysis of the major phyla of domain Bacteria also showed variation in the sequence frequencies between different classes, as well as variations in the vertical distribution within members of the same class. When considering Proteobacteria (Fig. 5), members of the class Alphaproteobacteria had the highest numbers. Their sequence frequencies increased in L2 relative to L1 and then exponentially decreased in L3 and L4. Conversely, the sequence frequency of Deltaproteobacteria increased with depth, and it had the highest sequence frequency in L4 (∼ 15% relative to the total bacterial sequences). Beta- and Epsiloproteobactreia had the least sequence frequency in the phylum Proteobacteria; additionally, their sequence frequencies were similar in all the mat layers investigated.

**Figure 6:**
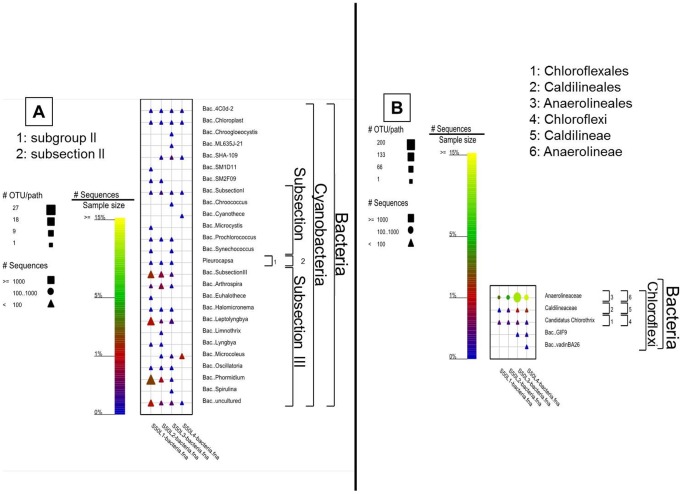
Graphical representation of the sequence frequency of the two main photosynthetic phyla (i.e., cyanobacteria (A) and Chloroflexi (B)) of the Domain Bacteria in different layers of Um Alhool mat. Members of the classes are shown also at the species level to facilitate a more specific sample comparison. The color of the symbol represents the relative frequency of the taxonomic path within the sample. The size of the symbol represents the number of OTUs at deeper phylogenetic levels within that taxonomic path. The shape of the symbol represents the number of sequences in the specific taxonomic path.

Overall, members of subsection III had the highest sequence frequency and were the most diverse cyanobacteria in Um Alhool mat. All cyanobacteria were more dominant in the uppermost layer compared with deeper layers (Fig 6A) except for *Chroogleocystis* and *Chroococcus* together, and *Cyanothece* that appeared only in L3 and L4. Contrarily, *Mircocysti*s and SM1D11 were only found at the uppermost layer. *Microcoleus* sp. was detected starting from L2 and it had the highest sequence frequency in L4. Subsection III, *Leptolyngbya* sp., and *Phoramidium* sp. not only had the highest sequence frequencies at the mat surface, but also were the most diverse, whereas their presence and diversity in the deeper layers were much less.

Chloroflexi had generally more sequence frequency in the two deepest layers (L3 and L4), as conditions were more suitable for them than in the upper layers. Members of family Anaerolineaceae were the most abundant between all detected members of phylum Chloroflexi in the mat (Fig. 6B).

#### 2. Other communities

**Figure 7:**
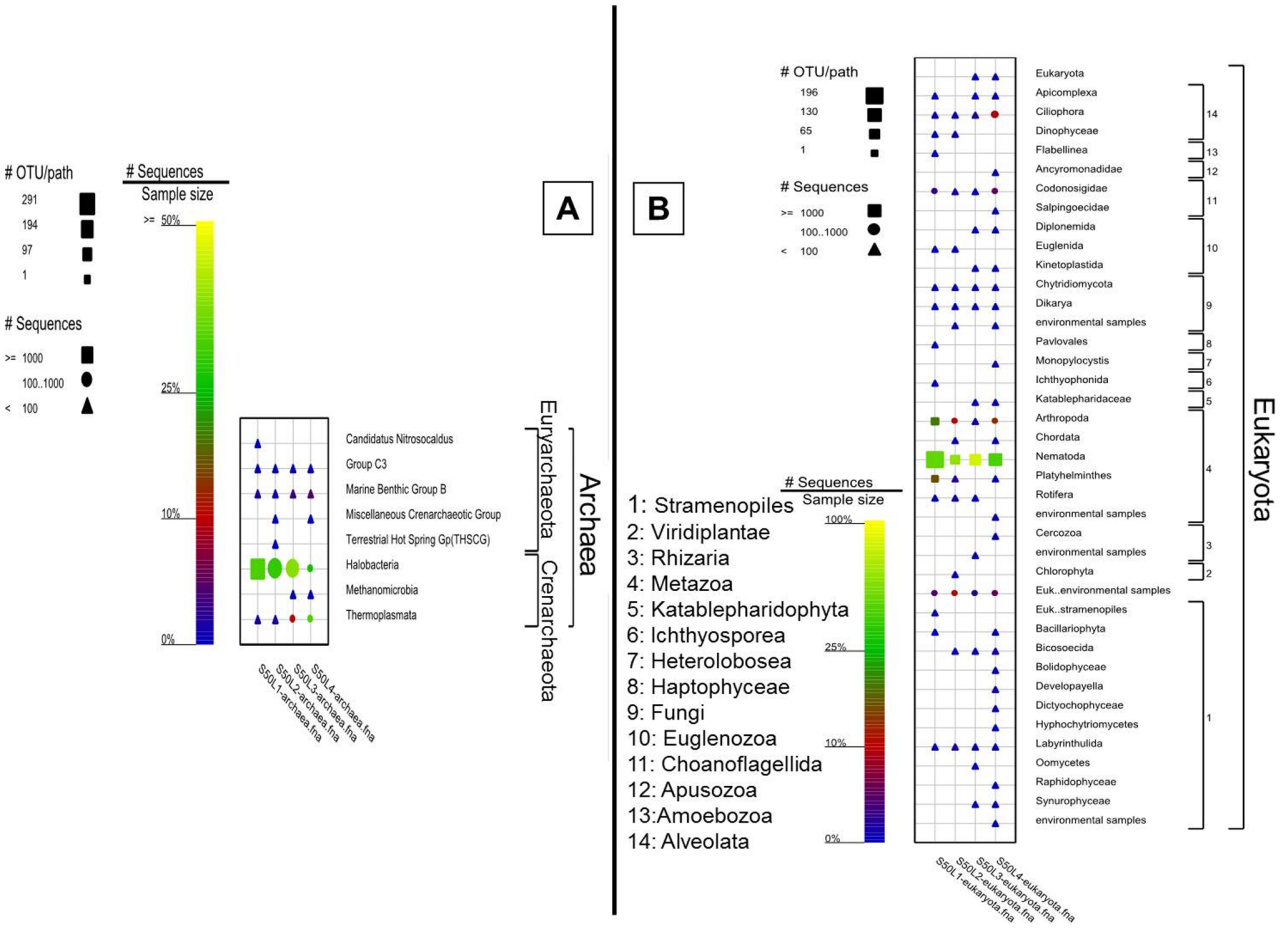
Graphical representation of the sequence frequency in the studied mat, showing major detected classes within the Archaeal and Eukaryotic domains. The symbols and sample naming are explained in detail in Fig. 6. Note different legends for OTU/path for each panel, and scale-bars for relative sequence frequency for several combined panels.

2.1. Archaeal community analysis: Members of phylum Crenachaeota dominated the whole Archaeal domain in all layers of the studied mat (∼ 92%–97%) compared with members of Euryarchaeota (∼ 3–8%) (Fig. 7A). Overall, members of genus Halobacteria were the most abundant and the most diverse group. However, this group exhibited vertical gradients through all layers of the mat, ranging from having the highest sequence frequency and being most diverse in the uppermost surface, to the least in the deepest layer (L4). Members of group 3 and Marine benthic group B of the phylum Euryarchaeota were found to have similar sequence frequencies and diversities in all the layers of the mat.

2.2 Eukaryotic community analysis: In addition to bacterial and archael community analysis, we investigated the eukaryotic community structure in Um Alhool mat (Fig. 7B). The mat was inhabited by several eukaryotic organisms belonging to the phyla Alveolata, Choanoflagellida, Euglenozoa, Fungi, Katablepharidophyta, Metazoa, Viridiplantae (chlorophyta), and stramenopiles (Labyrinthulida and Bacillariophyta) in addition to several unknown environmental organisms. Members of the phylum Metazoa were the most dominant in the eukaryotic community of the mat. Within this phylum, the highest sequence frequency was from members of the class Nematoda (45%–87%), followed by Arthropoda (∼ 1%–20%) and class Platyhelminthes (∼ 2% and 16%; found just in two layers). Nematodes appeared at the surface, but their presence was highest in L3 and L2, and it was the least in L4. Arthropods were found in all layers, but their presence was highest in the uppermost layer. Although Fungi were found in all layers, their sequence frequency was very low compared with the whole investigated eukaryotic community. Members of Bacillariophyta were detected in very low numbers in L1 and L4, and they were below the detection limit in the middle layers.

## Discussion

Um Alhool mat is a very interesting self-sustaining microbial mat. It is an active and highly dynamic ecosystem that harbors a diverse array of microorganisms interacting with each other as well as with their environment. In spite of no surface sea water supply, the mat ecosystem is permanently covered with saline water in winter that seeps from below, which comes from adjacent Gulf water, in addition to limited freshwater precipitations during winter season (55.8 mm in 2011). Water chemistry analysis (Table 1) demonstrated nutrient availability for photosynthetic microorganisms, but their concentrations are less than that of other seawater [32]. In spite of that the mat showed rates of photosynthesis very similar to other microbial mats. This indicates that Um Alhool ecosystem is highly efficient in remineralizing the organic material synthesized in the euphotic zone, which will, in turn, provide nutrients for the photosynthetic microorganisms in the euphotic zone of the mat ecosystem.

Other nutrient resources such as dust deposition, river discharges, and nitrogen fixation by cyanobacteria cannot be ignored. The studied mat is located in an area that it is away from any human or industrial activities. Additionally, there are no rivers in the region that might provide nutrient-rich water to supply the mat system that is permanently surrounded by sand dunes. Therefore, nutrient content of the mat is, most probably, affected finitely by surrounding nutrients supply such as dust storms that occur in the summer season. The measurements in the current study were performed in winter; subsequently, the dust contribution to the nutrients in the mat was not determined. Nitrogen fixation by cyanobacteria is an important N-source for the microbial mat; however, its exact contribution was not determined in this study.

The mat surface reflected some incident irradiances, whereas the majority (∼ 98.8%) was absorbed in the ecosystem by photopigments (see Fig.2), detritus, and other colored compounds in the euphotic zone [13]. Incident irradiance is most likely utilized efficiently in the upper layers of the mat as the PAR spectra exponentially attenuated reaching 1% at 1.7 mm (Fig. 3C). Interestingly, the attenuated incident irradiance showed enhanced local absorptions at 3 depths inside the mat at 0.5, 0.8, and 1.2 mm (Fig. 3C). The first two (at 0.5 and 0.8 mm) correspond to zones of higher photosynthetic activities (Fig. 3B) as shown from the light/dark shift measurements. These zones could be due to either the presence of high numbers of photosynthetic cells or high Chl *a* content. Indeed, the hyperspectral imaging of the Chl *a* signal (MPBI) in the upper layer of the mat (Fig. 2B&C) demonstrated the presence of several Chl *a* patches found at different depths representing hotspots of active light absorption. However, the third enhanced absorption (at 1.2 mm) could be because of the inactive light absorption by abiotic components of the mat [13], as there was limited corresponding photosynthetic activity.

Efficient utilization of incident irradiance and the subsequent photosynthetic activity in the uppermost layer of the mat was supported by the spatial distribution of the pigments (Fig. 2E), microsensor measurements of photosynthesis (Fig. 3A&B), and the 454 pyrosequencing of the 16S and 18S rRNA data (Figs. 6A & 7B). The upper layer of the mat contained elevated levels of photopigments that contributed to PAR absorption such as Chl *a*, phycocyanin, and carotenoids. These pigments characterize the oxygenic photosynthetic microorganisms such as cyanobacteria, diatoms, and eukaryotic algae, which showed elevated sequence frequencies in the upper layer compared with the deeper ones (Figs. 6A & 7B). Additionally, the near infra-red radiation is absorbed in the deeper red layer (L3) (Fig 2D), where anoxygenic photosynthetic microorganisms (Chlorobia and Chloroflexi) were found in a very high sequence frequency (Figs. 4 & 6B). These types of photosynthetic microorganisms are capable of utilizing the near infra-red radiation as they contain bacetriochlorophylls (Fig. 2D&F), which is the primary pigment in their light harvesting apparatus.

Interestingly, Chl *a* was measured in the deeper layers inside the studied microbial mat (Fig. 2E). Photosynthetic microorganisms in the mats are periodically exposed to burial events resulting from discharging of the sediment load when the speed of the current slows down, or from dust storms. The motile cyanobacteria can migrate again to the upper layer; however, the others stay buried commencing their heterotrophic life form, but at the same time retaining their Chl a content preparing for better conditions. Similar phenomenon was described earlier in hypersaline microbial mats, where cyanobacteria located deep at 14 mm maintained their Chl *a*, and they were able to resume photosynthesis shortly after exposing them to light and oxygen [33].

Ideally in microbial mats, photosynthetic microbes that consistently dominate the upper 1–2 mm, where light penetrates, are the main source of energy, organic material, and O_2_ to the other components of the mat ecosystem. Heterotrophic microorganisms in different layers of the mat utilize the organic material using the series of terminal electron acceptors (TEA) based on their availability and the energy gain. The microbes, where O_2_ is available perform oxygenic respiration and in turn they gain the highest amount of energy. When O_2_ is consumed, nitrate respiration will start contributing more, followed by iron and manganese reduction. Considerable contribution to the organic materials oxidation in the microbial mats would come from sulfate reduction. In the deeper layers of the mat, the fermenters flourish and can be traced by their fermentation products such as organic acids and hydrogen. These products will serve as a suitable substrate for the methanogeic Archaea that contribute to the green-house gas emission.

Our results showed similar scenario, as the photoautotrophic communities (cyanobacteria, diatoms, and algae) of the mat showed high photosynthetic rates, similar to those of other microbial mats (for example: [13], [34], [35]; see Table S1 in File S1). Um Alhool mat ecosystem appears to be adapted to high light conditions as a 4-times increase in incident irradiance resulted in a 4-times increase in photosynthetic rate, suggesting that the mat system was not saturated. This behavior is expected because the mat experiences very high incident irradiance during the day (>1800 μmol photon m^−2^ s^−1^). Microorganisms in the mats exposed to such extreme environmental conditions are equipped with different protective mechanisms to adapt to this environment. They can tune their photopigment production and distribution; also they migrate away to deeper layers where they can utilize the available light to the highest possible efficiency.

On the other hand, the measured sulfate reduction rates in the mat were much less than those of the other microbial mats (for example: [36]–[39]). It has been estimated that sulfate reduction may contribute to more than 50% of total organic material degradation in microbial mats [40]. However, this is not the case in the upper centimeter in the studied mat, where the highly photosynthetically-active part of the mat showed more organic material degradation occurred due to aerobic respiration rather than sulfate respiration. Indeed, the areal respiration rates in the euphotic zone is almost 10-times higher than those in the integrated areal rates of sulfate reduction in the upper 1 cm of the mat (0.255 versus 0.028 μmol m^−2^ s^−1^), and is 1.5-times more than that of the upper 2 cm. The presence of a diverse eukaryotic community in the mat, particularly nematodes, (Fig 7B) considerably contributes to oxygenating the deeper layers of the mat through bioturbation [41]. In some cases, the measured sulfate reduction could be underestimated because of relatively long incubation of the sediment core, which might result in sulfide oxidation. In our case, the cores were tightly closed and incubated in dark, which weakened this possibility.

Overall, although Um Alhool mat is supplied by sea water from below, it has active carbon and sulfur biogeochemical cycling resulting in highly efficient mineralization/remineralization. We expect that nutrient availability in the studied mat ecosystem is a result of not only seawater but also because of efficient mineral recycling in the ecosystem.

Interestingly, sulfide disappeared between the depths of 2–4 cm in the mat, which could be because of sulfide oxidation through native transporters in the sediment. This phenomenon was discovered recently in the sediments, in which long filamentous bacteria transport electrons within the sediment over a distance of 1 cm [42]. Another possible reason could be bioturbation that helps in the formation of Fe (III) deep in the sediment, which in turn will oxidize sulfide. However, we have not deeply investigated this because it was not the focus of this study. Moreover, members of the methanogenic Archaea belonging to the order Methanosarcinales were found exclusively in the third and the fourth layers, and we did not investigate beyond these layers.

### Microbial community analysis

The oxygenic layers of the community were expected to be dominated by Cyanobacteria of the *Halothece*, *Spirulina*, and *Phormidium* types, but microscopy and molecular analyses showed that oxygenic phototrophs were outnumbered by the other constituents of the community, including chemotrophs and anoxygenic phototrophs. The microbial community structure of Um Alhool mat was dominated by Proteobacteria (Fig. 4), which is expected as it is the largest and most diverse of the bacterial kingdoms. Members of different classes belonging to this phylum (i.e., alpha, beta, and gamma) have diverse metabolic capabilities: some are phototrophic, but most are chemolithotrophs or chemoorganotrophs. Members of Alphaproteobacteria were most abundant in L2 (Fig. 5), as the conditions in L2 were suitable for the purple non-sulfur bacteria, which are anaerobic photosynthetic bacteria that have bacteriochlorophylls (Fig. 2F) to capture light energy. These ecologically resourceful microorganisms can also be chemotrophic when growing in the dark. Gammaproteobacteria were found in all layers but they had more sequence frequency in the upper layer as the members of this group are divided into two groups: the purple sulfur bacteria, which can be obligate anaerobes, and aerobic or facultative anaerobic bacteria. Deltaproteobacteria contains most of the known anaerobic sulfate- (*Desulfovibrio*, *Desulfobacter*, *Desulfococcus*, *Desulfonema*, etc.) and sulfur- (e.g., *Desulfuromonas* spp.) reducing bacteria [43], which explains the increasing sequence frequency of members along the depth (Fig. 5). This was also supported by the higher sulfate reduction rates in the deeper layers (Fig. 3D).

Members of domain Archaea had less sequence frequency, had lower diversity, and their distribution was less stratified than in *Bacteria*. Detected groups included organisms affiliated with the Methanosarcinales, the Halobacteriales, and uncultured groups of Euryarchaeota**.** In the studied mat, members belonging to Halobacterium were the most abundant and more diverse because they can tune their metabolism in response to changing in the environmental conditions. For example, in addition to their optimal growth under oxic conditions, they are capable for evolving a synchronized set of responses to adapt to oxygen limitation [44]. When conditions become more reduced, Halobacteria increase buoyancy and scavenge molecular oxygen, and under extreme anoxic conditions they shift to anaerobic respiration and fermentation [44].

Our results showed that the prokaryotic diversity exceeded that for Eukaryotes; a case that was also found in several other studies (e.g., [45]–[47]). This is mainly due to the ability of bacteria to develop strategies to adapt to ever changing environmental conditions and habitats throughout their life history [45]. While there have been large scale extinction events in eukaryotes, bacteria have remained relatively persistent and this is mainly because: (i) bacteria have versatile metabolic pathways, hence they can produce energy from wide range of organic and inorganic compounds. They have also a spectrum of electron acceptors that they can use such as, oxygen, nitrate, iron III, arsenic IV, selenium VI, etc. However, eukaryotes gain their energy from a limited range of reduced compounds and have very limited electron acceptors. (ii) Prokaryotes have a “feast or famine” mode of existence and can tolerate long periods of starvations lasting several months to years. They can achieve this because some prokaryotes can make marked alterations in their cellular ultra-structure, such as production of endospores or cysts. Others decrease in cell size, shrink their protoplast, in addition to altering their gene expression and changing their mode of life from planktonic to sessile (e.g., biofilm formation). (iii) Some bacteria evolved “pack rat” strategy by storing diverse reserved materials including glycogen-like polysaccharides, polyhydroxybutarate, polyphosphate, and sulfur to persist during famine conditions (reviewed in [48]).

## Supporting Information

File S1 SI file that contains detailed methodology describing sequence analysis, in addition to Table S1, Areal rates of photosynthesis in microbial mats, exposed to different incident intensities and temperatures. Figure S1, Rates of areal gross (red circle), net (black square) photosynthesis, and rates of respiration (blue triangles), at different incident irradiances.
(DOCX)
