# Supplementary material for: Community Structure and Activity of a Highly Dynamic and Nutrient-Limited Hypersaline Microbial Mat in Um Alhool Sabkha, Qatar
Source: PLoS One. 2014 Mar 21;9(3):e92405. doi: 10.1371/journal.pone.0092405 (PMC3962408; doi:10.1371/journal.pone.0092405)
Supplement: File S1 — SI file that contains detailed methodology describing sequence analysis, in addition to Table S1, Areal rates of photosynthesis in microbial mats, exposed to different incident intensities and temperatures. Figure S1, Rates of areal gross (red circle), net (black square) photosynthesis, and rates of respiration (blue triangles), at different incident irradiances. (DOCX) [file pone.0092405.s001.docx]

Supplementary Information

**Community structure and activity of a highly dynamic and nutrient limited hypersaline microbial mat in Um Alhool Sabkha, Qatar**

Roda Al-Thani, Mohammad A. A. Al-Najjar, Abdul Munem Al-Raei, Tim Ferdelman, Nguyen M. Thang, Ismail Al Shaikh, Mehsin Al-Ansi and Dirk de Beer

## Materials and Methods:

**Sequence Analysis**

Diversity and community structure analyses were performed on 18078 Bacterial, 9299 Archaeal, and 22651 Eukaryotic sequences obtained from different layers of the studied mat. Sequence analysis was done as described in Pruesse et.al. [[1](#_ENREF_1)]. Briefly, sequence reads from amplicon pyrosequencing were aligned and quality-controlled by the bioinformatics pipeline of the SILVA rRNA gene database project [[2](#_ENREF_2)]. Alignment of all reads was performed using the SILVA Incremental Aligner against the SILVA SSU rRNA seed [[1](#_ENREF_1)]. Non-aligned reads (i.e., putative contaminations or artifacts), as well as the ones with reads shorter than 50 aligned nucleotides and reads with more than 2% of ambiguities or 2% of homopolymers, respectively, were removed. The filtered datasets were de-replicated, clustered and classified in parallel on a sample by sample basis. De-replication (identification of identical reads ignoring overhangs) and clustering (OTU definition based on a non-redundant subset of reads) was done using cd-hit-est (http://www.bioinformatics.org/cd-hit) applying identity criteria of 1.00 and 0.98, respectively, both times with a word size of 8. For each OTU/cluster, the longest read was then used as a reference of this cluster for taxonomic classification. The classification was performed by a local nucleotide BLAST search against the non-redundant version of the SILVA SSURef dataset (release 106; http://www.arb-silva.de) using blast -2.2.22+ <http://blast.ncbi>. nlm.nih.gov/Blast.cgi) with standard settings. To filter out low identity and artificial BLAST hits, hits for which the function (%sequence identity +%alignment coverage)/2, did not exceed the value of 93.0 were discarded. For the analyzed reads with sufficiently good BLAST hits, the taxonomic classification of the best BLAST hit according to the SILVA taxonomy has been assigned to the read. Reads without any BLAST hits, or reads with weak BLAST hits only, were classified as ‘No Relatives’. Finally, the taxonomic path of each cluster reference was mapped to all reads within the corresponding cluster as well as to their corresponding replicates.

This last step allowed to obtain quantitative information (number of individual reads representing a taxonomic path), within the bounds of PCR and pyrosequencing biases. To confirm the taxonomic affiliation of the sequences, all cluster references were imported into ARB [[3](#_ENREF_3)] and inserted into the guide tree of the SILVA SSURef dataset (release 108).

Table S1 Areal rates of photosynthesis in microbial mats, exposed to different incident intensities and temperatures.

|  | Incident | areal P_G_ | Type/Condition | Source |
| --- | --- | --- | --- | --- |
|  | µmol photon m^-2^s^-1^ | µmol O_2_ m^-2^s^-1^ |  |  |
| 1 | 75 | 1.8 | cyanobacterial mat, 15°C | Epping &Kühl, 2000 |
| 2 | 70 | 1.6 | artificial mat, 5°C | Buffan-Dubau et al, 2001 |
| 3 | 125-1450 | 0.35-2.5 | Hypersaline mat, Chiprana, Spain | Jonkers H.M. et al, 2003 |
| 4 | 300 | 1.2-2.4 | Hypersaline mat, Chiprana, Spain, Nutrients effect | Ludwig et al., 2006 |
| 5 | 425 | 2.2-3.6 | Hypersaline mat, Solar lake, Egypt | Wieland&Kühl, 2000 |
| 6 | 43-350 | 0.1- 0.72 | Microbial mat, light adaptation | Wieland et al, 2003 |
| 7 | 1827 | 3.2 | hot spring microbial mat | Ferris et al, 2003 |
| 8 | 50 | 0.9-2.4 | Solar lake mat, T effect 25-40°C, Max. Φ | Wieland&Kühl, 2000 |
| 9 | 1000 | 1 | microbial mat, Spain | Martinez-Alonso et al. 2004 |
| 10 | 20-212 | 0.9-3.0 | hypersaline mat, Exmouth gulf in Australia | Al-Najjar et. al., 2012 |
| 11 | 30-500 | 1.2-3.2 | hypersaline mat, Saadeyat island Abu-Dhabi | Al-Najjar et. al., 2010 |

Fig. S1. Rates of areal gross (red circle), net (black square) photosynthesis, and rates of respiration (blue triangles), at different incident irradiances.

1. Ionescu D, Siebert C, Polerecky L, Munwes YY, Lott C, et al. (2012) Microbial and Chemical Characterization of Underwater Fresh Water Springs in the Dead Sea. PLoS ONE 7: e38319.

2. Pruesse E, Quast C, Knittel K, Fuchs B, Ludwig W, et al. (2007) SILVA: a comprehensive online resource for quality checked and aligned ribosomal RNA sequence data compatible with ARB. Nucleic Acids Research 35: 7188–7196.

3. Ludwig W, Strunk O, Westram R, Richter L, Meier H, et al. (2004) ARB: a software environment for sequence data. Nucleic Acids Research 32: 1363-1371.
